# Bacterial Contamination and Antimicrobial Resistance in Two-Spotted (*Gryllus bimaculatus*) and House (*Acheta domesticus*) Cricket Rearing and Harvesting Processes

**DOI:** 10.3390/vetsci11070295

**Published:** 2024-07-01

**Authors:** Jamlong Mitchaothai, Nils T. Grabowski, Rachakris Lertpatarakomol, Tassanee Trairatapiwan, Achara Lukkananukool

**Affiliations:** 1 Office of Administrative Interdisciplinary Program on Agricultural Technology, School of Agricultural Technology, King Mongkut’s Institute of Technology Ladkrabang (KMITL), Bangkok 10520, Thailand; 2 Institute for Food Quality and Food Safety, University of Veterinary Medicine Hannover (TiHo), 30173 Hannover, Germany; nils.grabowski@tiho-hannover.de; 3 Faculty of Veterinary Medicine, Mahanakorn University of Technology (MUT), Bangkok 10530, Thailand; rachakris@gmail.com (R.L.); tassanee@mut.ac.th (T.T.); 4 Department of Animal Production Technology and Fisheries, School of Agricultural Technology, King Mongkut’s Institute of Technology Ladkrabang (KMITL), Bangkok 10520, Thailand; achara.lu@kmitl.ac.th

**Keywords:** bacterial contamination, antimicrobial resistance, two-spotted cricket (*Gryllus bimaculatus*), house cricket (*Acheta domesticus*), rearing and harvesting processes

## Abstract

**Simple Summary:**

The farming of edible crickets (both two-spotted and house crickets) is increasing. Safety and sustainability in the production of crickets are crucial for consumers and farmers. *Klebsiella* spp. and *Enterobacter*, classified as non-pathogenic bacteria, are often found. For studied pathogenic bacteria, *E. coli* was detected at a lower rate of 5%, while *Salmonella* spp. were not detected. The antimicrobial resistance of isolated *E. coli* mainly involves penicillin G, amoxicillin, ampicillin, erythromycin, lincomycin, and tiamulin. Good sanitary practices, e.g., cleaning and remaining dry, as well as boiling crickets during the harvesting process, may be helpful for the safety of edible cricket production.

**Abstract:**

Food safety for cricket production is a crucial factor in producing edible crickets with safety for consumers and sustainability for two-spotted (*Gryllus bimaculatus*) as well as house (*Acheta domesticus*) cricket production. This study was conducted by simultaneously rearing two cricket species, comprising two-spotted crickets (*G. bimaculatus*) and house crickets (*A. domesticus*). A total of 16 rearing crates were used for the present study, which were allocated into 8 rearing crates for each studied cricket species, including paper egg cartons. Cricket eggs were incubated in the rearing crates. Once the crickets hatched, tap water and powdered feed were provided ad libitum throughout the experiment. At the end of this study (35 and 42 days for the two-spotted and house crickets, respectively), all crickets were harvested, rinsed in tap water, and boiled in water for 5 min. During the rearing and harvesting processes, samples were collected from various potential contamination points for bacteria, including *E. coli* and *Salmonella* spp. There were samples of the initial input (feed, drinking water, and staff hands), rearing environment (water pipe, crate wall, living cartons, frass, and cricket surface), and harvesting crickets (harvested, washed, and boiled crickets), with a 2-week sampling interval, except for the last round of sampling for the two-spotted crickets. Subsequently, all samples were submitted to isolate and identify contaminated bacteria. The samples from the last round of sampling for both kinds of crickets were submitted to quantify the level of contamination for *E. coli* and *Salmonella* spp., including antimicrobial resistance by the disk diffusion method for the positive isolate. The results showed that bacterial contamination was found in the rearing of both cricket species, primarily involving *Klebsiella* spp. and *Enterobacter* spp., mainly found in prepared drinking water and the water pipes of drinking water supply equipment, which are potential sources of contamination with cricket frass. *E. coli* was found in 4.8% and 4.3% of the two-spotted and house crickets, respectively, while no presence of *Salmonella* spp. was detected in any submitted samples. The quantification of *E. coli* and *Salmonella* spp. indicated *E. coli* contamination near the water pipe and the frass of two-spotted crickets, but *Salmonella* spp. was undetectable in both two-spotted and house crickets. The antimicrobial resistance of isolated *E. coli* mainly involved penicillin G, amoxicillin, ampicillin, erythromycin, lincomycin, and tiamulin. Thus, good farm management with proper sanitation practices (such as cleaning and keeping the environment dry), as well as boiling crickets during the harvesting process, may help ensure the safety of edible cricket production.

## 1. Introduction

It is commonly known that edible insects have nutritional benefits for consumers. Edible insects have additional benefits such as less land use for rearing, a high rate of reproduction, and high feed conversion efficiency [1]. Furthermore, edible insects have also been promoted by various organizations and governments as a way of taming climate change, conferring the environmental benefit of reduced greenhouse gas emissions [1]. These pressures increasingly affect livestock production. In addition, sustainability in animal-based protein production is receiving increased attention. The conditions for insect farming (particularly housing, feed contaminants, feeding, animal health, and animal welfare) are crucial factors for ensuring food safety and sustainability [2]. The edible insect supply chain consists of (1) the rearing process, (2) sorting/harvesting, (3) post-harvesting and processing, and (4) distribution and storage [3]. Many aspects of food safety should be evaluated for edible insect production. For instance, feed substrates can be sources of microbiological and chemical threats to the supply chain through harmful bacteria, heavy metals, viruses, mycotoxins, and prions [3,4]. Fernandez-Cassi et al. [4] reported the importance of allergenicity in edible insects, including crickets.

Cricket farming for mass production is an increasingly important alternative in terms of using animal protein as a novel food source [5]. Both two-spotted (*Gryllus bimaculatus*) and house (*Acheta domesticus*) crickets are the main species used in production as well as research. Increasing cricket farm numbers would lead to higher variation in cricket production chains, with probably different standards in production. Literature reviews by Fernandez-Cassi et al. [4] as well as this study found very little information about the farming environment, and very few studies have described the actual conditions used for cricket rearing, which have an impact on microbial counts, microbial populations, and chemical composition. According to the report by Fernandez-Cassi et al. [6], the high Enterobacteriaceae counts in fresh samples of house crickets are classified as *Klebsiella* spp., *Citrobacter* spp., and *Enterobacter* spp. These bacteria are gut microbes that possibly contribute to bacterial contamination in the cricket production chain. In principle, good practices in related fields, such as Good Hygiene Practices (GHPs), Good Agricultural Practices (GAPs), and Good Veterinary Practices (GVPs), should also be applied to cricket production like in conventional farm animal production [4]. Antimicrobial resistance (AMR) is a major issue for the health of both humans and animals, particularly in food-producing animals that provide nourishment for consumers. This is an important factor for food safety. However, little information exists about antimicrobials in cricket products [7,8]. Due to the limited amount of useful information available concerning edible cricket farming, the current study was conducted to explain and gain more insight into bacterial contamination and antimicrobial resistance in two-spotted and house cricket rearing and harvesting processes.

## 2. Materials and Methods

### 2.1. Cricket Rearing and Management

To determine and quantify the bacterial contamination in the production chains of two-spotted (*G. bimaculatus*) and house (*A. domesticus*) crickets, the crickets were reared at a farm facility belonging to the Animal Science Division, Department of Animal Production Technology and Fisheries, School of Agricultural Technology at King Mongkut’s Institute of Technology Ladkrabang, Thailand. To prepare rearing *Acheta domesticus* and equipment, a quaternary ammonium compound was applied. The crickets were reared in an open barn with an environmental temperature of 32.03 ± 1.33 °C and a relative humidity of 72.35 ± 8.46%. The details of cricket rearing and management followed an earlier report [5] with minor adaptation, except for half the size of the rearing cricket crate and approximately half the quantity of cricket eggs applied for both types of studied crickets. Briefly, the rearing cricket crate wall and floor were made from polycarbonate, and egg board cartons were added to each rearing crate to provide a living place for crickets. Cricket eggs of the two-spotted and house variety were purchased from a commercial cricket breeder in Thailand. A commercial cricket feed (Pure Pride^®^; PP feed, the TFMS (Saraburi) Co., Ltd., Sao Hai, Thailand) containing 21.15% crude protein (CP) and 4.07% crude fat was used. Each rearing crate was equipped with a plastic feeding tray that had a rough surface provided on the top of the cartons. A total of 16 rearing crates were used for the present study, with 8 rearing crates (8 experimental units) allocated for each type of studied crickets. The feeding regime was ad libitum by adding a feeding tray and an amount of feed proportionally to the larger size of crickets. The feed for feeding the crickets was stored in two plastic buckets (size of 120 L of each) with a tight cover. Tap water was prepared for the crickets as drinking water by putting it in two plastic buckets (60 L each) and allowing for the vaporization of chlorine for 3 days in advance with a mesh cover and then covering tightly. Water was provided through a PVC pipe (rectangle shape) of approximately 50 cm in length and 3.81 cm in diameter with 22 holes on its rough surface filled with thread to allow the crickets to sip water through the thread. After hatching nymphs, Pure Pride^®^ cricket feed was offered to the nymphs or crickets throughout the experiment, every day, by mixing the leftover feed with the newly offered feed. A small sweeper was used to clean the cricket crates daily and collect frass with dirt from the floor of the cricket crates. There was a set of cleaning equipment for each cricket crate. The staff taking care of the crickets washed their hands before starting work in the cricket barn and after finishing work for each cricket rearing crate. When the crickets had reached more than 95% full growth based on the appearance of wings, the rearing stage ended. A few days before the end of each batch, a tray with a moisturized mixture of autoclaved coconut flakes and dust was offered, allowing some female crickets to lay eggs. Subsequently, the feed was withdrawn at 24 h before harvesting the crickets. After harvesting, all crickets were killed by freezing them in a cricket bag with ice for 15 min until death, which is a method that complies with animal welfare. The crickets were then rinsed in tap water three times and boiled in water for 5 min. The crickets were subsequently placed on a mesh shelf to allow water to drain and the crickets to dry for approximately 10 min.

### 2.2. Sample Collection

**Figure 1:**
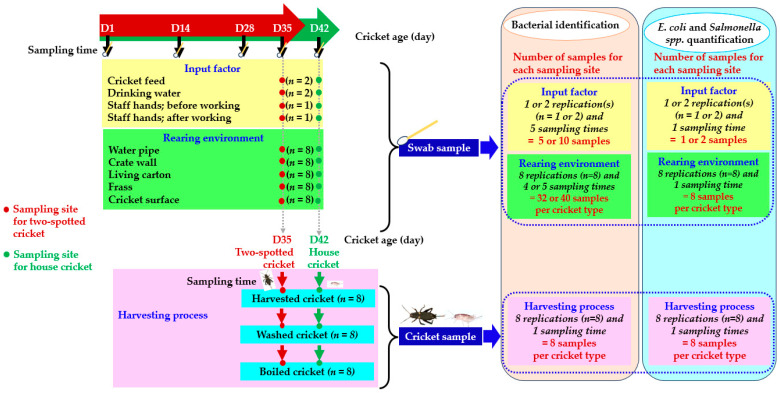
Sampling time, sample type, and sampling site for the current study. There were starting times on the first day (D1; the first day of hatching) and sampling times on day 1 (D1), 14 (D14), 28 (D28), 35 (D35), and 42 (D42) of cricket age. The ends of the cricket rearing were 35- and 42-day-old two-spotted and house crickets, respectively.

To measure bacterial contamination in the production chains of two-spotted and house crickets, critical points and sources for the bacterial contamination were determined, as demonstrated in Figure 1. For swab sample collection, a sterile cotton swab was soaked with a sterile normal solution, which was then used to swab a specific surface site. Each surface site was swabbed in a 25 cm^2^ area, after which the swab samples were placed into transport media. All swab samples were stored at 3–5 °C and then submitted to a microbiological laboratory within 24 h. For the swab samples of the feed, water, and hand, nearly the same position was swabbed for each sampling time. In the meantime, a marked specific area of 25 cm^2^ for the swab sample of the water pipe, crate wall, and living carton was labeled to ensure the swabbing of the same position area at each sampling time. To swab frass samples, around 25 cm^2^ of the area surrounding the center of the frass pile on the crate floor was selected for swabbing, except for the sampling at D1, where an area of the crate floor was swabbed as no frass appeared on the first day. There was a very small-sized nymph at hatching; the newly hatched nymphs were decoyed to move into a sterile plastic bag via a coarse surface and rather hard paper, and then approximately a 25 cm^2^ area of cricket mass in the bag was swabbed. For 14-day-old and older nymphs, a random selection of crickets in each crate was used to obtain swabs, covering a collective area of about 25 cm^2^. There were 4 and 5 rounds of sampling for the two-spotted and house crickets, respectively. At the end of cricket rearing, swabs and cricket samples were collected twice for additional testing of quantification for *E. coli* and *Salmonella* spp. levels at all sampling sites. To avoid repeated and biased positions in the swabbing of these double samples, a 25 cm^2^ area nearby unmarked and marked sampling sites for swabbing was used. For cricket samples in the harvesting process, approximately 55 g of a cricket sample at each sampling site was first collected and kept in a thick plastic zip bag, after which 25 g of this sample was allocated and transferred to another zip bag as a sample for quantifying *E. coli* and *Salmonella* spp. levels. The sampling technique for collecting cricket samples included the sum of subsamples from 5 positions (at 4 angles and 1 center of a supposed square) of cricket mass in a container for harvesting, which was randomized, collected, and then kept in a thick plastic zip bag using 25 g crickets per sample. Biomass productions obtained from the present study were 1.099 ± 0.232 and 0.909 ± 0.320 kg per rearing crate for the two-spotted and house crickets, respectively.

### 2.3. Microbiological Isolation and Analysis

Swab samples from the studied surface site and cricket samples were cultured for possible bacterial contamination by following Section 1 of *Clinical Veterinary Microbiology* (2nd edition) [9]. Then, each bacterial isolate was identified by a biochemical test according to the Laboratory Procedures in Clinical Veterinary Bacteriology [10]. To quantify the contamination levels of *E. coli* and *Salmonella* spp., a set of swab samples from the studied surface site and cricket samples for the level of contamination for *E. coli* and *Salmonella* spp. was created by AOAC official method 991.14 [11] and BIO-RAD chromogenic method [12], respectively.

### 2.4. Antimicrobial Susceptibility Test of E. coli and Salmonella spp.

The Kirby–Bauer method (disc diffusion method) was applied to conduct susceptibility testing of isolates for *E. coli* and *Salmonella* spp. to a panel of antimicrobial agents. The antimicrobials in the test panel were chosen based on common use for livestock animals in the past and the present [13], including antimicrobial agents based on earlier reports for resistance in insects [4,8,14,15,16]. The test panel included penicillin G (PNG, 10 µg), amoxicillin–clavulanic acid (AMC, 30 µg), amoxycillin (AMX, 10 µg), ampicillin (AMP, 10 µg), cefalexin (CEF, 30 µg), colistin (COL, 10 µg), oxytetracycline (OTC, 30 µg), doxycycline (DOX, 30 µg), enrofloxacin (ENR, 5 µg), halquinal (HAL, 30 µg), trimethoprim–sulfamethoxazole (SXT, 25 µg), neomycin (NEO, 30 µg), gentamicin (GEN, 10 µg), lincomycin (LIN, 15 µg), erythromycin (ERY, 15 µg), and tiamulin (TIA, 30 µg). Antimicrobial resistance breakpoints were interpreted following the criteria from the Clinical and Laboratory Standards Institute [17].

### 2.5. Statistical Analysis

The Chi-square test was used to compare the proportions of bacteria isolated and contaminated both within and between the experimental field and house crickets by using R version 4.3.1 (R core Team, 2023).

## 3. Results

### 3.1. Bacterial Isolation

There were 20 swab samples taken from sampling sites for the input factor (Table 1). The number of samples from the two-spotted crickets included 160 swab samples for the rearing environment and 24 cricket samples for the studied two-spotted crickets (Table 1). On day 35 of cricket rearing, there was an accident involving some two-spotted crickets falling into a rearing crate of the house crickets, which resulted in the mixing of these two cricket types; these were excluded from the study on day 42 of the experimental trial. Thus, the total number of samples from the house crickets was 195 swab samples for the rearing environment and 21 cricket samples (Table 1).

From the submitted swab and cricket samples for bacterial culture, bacterial isolation found *Achromobacter* spp., *Citrobacter* spp., *Enterobacter* spp., *Klebsiella* spp., *Pasteurella aerogenes*, *Proteus* spp., *Pseudonomas aeruginosa*, *Escherichia coli*, and *Salmonella* species. There were no bacterial isolates from the cricket feed or the hands of staff before working at the cricket barn (Table 1). Six samples (from ten swab samples) of drinking water were contaminated with bacteria, while the rest of the drinking water samples had no bacterial isolate at the first and the last round of swab sampling (no information provided). One sample (from five swab samples) of the staff hands after working revealed the contamination of *Achromobacter* spp. in the last round of swab sampling, whereas no bacterial isolation was found in the rest of the samples. The proportion of microbials isolated from submitted samples was not different (*p* > 0.05) between the two studied cricket species. For the percentage of the bacteria isolated to the submitted samples, there were some samples found more often than one bacterium species or type, resulting in high values of the percentage. For the rearing environment of both two-spotted and house crickets, there was an obvious contamination of *Klebsiella* spp. (Table 1) at the highest level (*p* < 0.05) in terms of the percentage (72.6% and 63.6%, respectively) among all bacteria isolated. The second highest (*p* < 0.05) bacterial contamination for the rearing environment was *Enterobacter* spp. for both two-spotted and house crickets (19.2% and 16.0%, respectively). Both the *Klebsiella* spp. and *Enterobacter* spp. proportions detected were not different (*p* > 0.05) between the two studied cricket species. The proportions of *E. coli* detected from the rearing environment were 4.8% and 4.3% for the two-spotted and house crickets, respectively, which were not different in the proportion between the two cricket species (*p* > 0.05). The contamination of *Klebsiella* spp. on crickets during the harvesting process showed the highest values of 87.5% (21 out of 24) and 85.7% (18 out of 21) for the two-spotted and house crickets, respectively (Table 1).

The presence or absence of bacterial isolates at each sampling site comprised the criteria for classification as “contaminated crate” and “uncontaminated crate”, respectively. The results of this classification for both studied cricket types are demonstrated in Table 2. It was found that the water pipe sites for all studied crates were contaminated with bacteria at the start (day 1) and at every sampling time until the end of cricket rearing (crickets aged 35 and 42 days for two-spotted and house crickets, respectively). The results of bacterial contamination proportion for the frass site were similar to those of the water pipe sites, except for no bacterial contamination found at the start (day 1). For the sites of the crate wall, living cartons, and cricket surface, there was an absence of bacterial contamination at the start, which gradually increased (*p* < 0.050) in terms of the proportion of bacterial contamination with cricket age, except for a significant proportion (*p* < 0.050) of bacterial contamination on the living cartons’ site of two-spotted crickets at day 14 onward (Table 2). For cricket samples in the harvesting process, there was no influence (*p* > 0.050) of washing and boiling on the proportion of bacterial contamination, although its values reduced by approximately 40% [100 − (62.5 + 57.1)/2 = 40.2] (Table 2) after boiling for both studied cricket species.

In the current study, 15 isolates of *E. coli* were detected, while Salmonella spp. were not detected (Table 1). From Table 3, the distribution of *E. coli* detected on the sampling site and at cricket age is illustrated. Approximately half of *E. coli* isolations (53.3%; 8 out of 15 isolations) originated on the water pipe. This is significantly higher (*p* < 0.05) than the proportion of *E. coli* isolates found at the crate wall and the frass, but not different (*p* > 0.05) from those found at the living cartons, and cricket surface.

### 3.2. Contamination Levels of E. coli and Salmonella spp.

The tests for the contamination levels of both *E. coli* and *Salmonella* spp. were <20 colony-forming units (CFUs) per cm^2^ and <10 CFU/g for swab and cricket samples, respectively. The swab area and cricket weight for each sample were 25 cm^2^ and 25 g, respectively. Therefore, the contamination of <20 CFU/cm^2^ and <10 CFU/g would be interpreted as no detection found in 25 cm^2^ and 25 g for the swab and cricket samples, respectively. For the *E. coli* contamination level in the two-spotted cricket rearing, there was 40 CFU/cm^2^ detected in one sample swabbed from the water pipe, and two samples from the frass site contained 120 and 20 CFU/cm^2^ for each (Table 4). The rest of the swab and cricket samples for the two-spotted crickets were undetected for both *E. coli* and *Salmonella* spp. (Table 4). The swab and cricket samples from all sampling sites of the house crickets were negative for both *E. coli* and *Salmonella* spp. (Table 4).

### 3.3. Antimicrobial Resistance of E. coli

For *E. coli* (Table 5), there was common (15 isolates out of 15 isolates) resistance to penicillin G, amoxycillin, ampicillin, lincomycin, erythromycin, and tiamulin. Resistance to cefalexin, oxytetracycline, and halquinol was found in one or two isolates. Meanwhile, *E. coli* was susceptible to amoxicillin–clavulanic acid, colistin, doxycycline, enrofloxacin, sulfamethoxazole–trimethoprim, neomycin, and gentamicin.

Antibiograms of *E. coli* were classified into four patterns (Table 6) comprising PNG + AMX + AMP + LIN + ERY + TIA, PNG + AMX + AMP + LIN + ERY + TIA + CEF, PNG + AMX + AMP + LIN + ERY + TIA + OTC, and PNG + AMX + AMP + LIN + ERY + TIA + HAL. The most common pattern (11 out of 15) of antibiograms was PNG + AMX + AMP + LIN + ERY + TIA, with 5–7 numbers of resistant antimicrobial agents.

## 4. Discussion

The production of both two-spotted and house crickets in this study was conducted under open barn conditions. There was a rather shorter duration for the rearing of the two-spotted (35 days) and house (42 days) crickets when compared to earlier reports by Mitchaothai et al. [5] (41.0 ± 1.0 days) and Bawa et al. [19] (49.0 ± 0.04 days). This can be explained by the higher environment temperature for house [20] and two-spotted [21] crickets, which resulted in the higher growth rates of the crickets. From the study of Magara et al. [21], the shortest adult longevity of two-spotted crickets was between 35 and 37 °C of environmental temperature. Further, countries located near equatorial, tropical, and subtropical regions tend to have optimal conditions for two-spotted cricket farming, with an average of 4–5 production cycles per year.

From the results of the bacterial isolation, there were two classes of bacteria isolated, including (1) opportunistic bacteria for humans, i.e., *Achromobacter* spp. [22], *Citrobacter* spp. [23], *Enterobacter* spp. [24], *Klebsiella* spp. [25,26], *Pasteurella aerogenes* [27], *Proteus* spp. [28], and *Pseudonomas aeruginosa* [29], and (2) pathogenic bacteria causing food-borne disease in humans, i.e., *E. coli* (pathogenic strains) and *Salmonella* spp. [30,31]. In the current study, the isolates of *E. coli* were not characterized, because it was not within the objectives of this study. In view of the different strains and further implications for public health, further studies should be conducted, including an investigation into the potential risk factors associated with *E. coli* contamination from cricket rearing. The same is true for other microbiological findings in this study. From the results of this study, the main opportunistic bacteria for humans were *Klebsiella* spp., followed by *Enterobacter* spp., across all three main production processes: the input factors, the rearing environments, and the harvesting of crickets (Table 1). Drinking water was a potential source of *Klebsiella* spp. and *Enterobacter* spp. contamination, although the source (day 1) could not be identified. This might be explained by the fact that drinking water was prepared to allow for chlorine vaporization for 3 days, resulting in the lowered efficacy of disinfectant properties and allowing a higher chance of microbial environmental contamination. In the meantime, one isolation of the sampling site for staff hands after working (*Achromobacter* spp.) at the last round of swab sampling indicated the spread of *Achromobacter* spp. only in the final stages of cricket rearing. This would result in very low chances of cross-contamination from the hands of the working staff. From the results of the rearing environment (Table 2), the site of the water pipe, i.e., the drinking water supply equipment, was a harbor of bacterial contamination. The equipment was made from a 3.81 cm diameter PVC pipe (rectangle shape) with 10–12 holes on its rough surface filled with thread to allow the crickets to sip water through the thread. Moisture from the drinking water and frass accumulated near the water pipe, which could create favorable conditions for bacterial growth. Biofilms are ubiquitous sources of microbial contamination that affect all areas of animal production, processing, and the food derived from them. Insect farm-related biofilms may be different from other livestock farm biofilms. The authors hypothesize that each species’ farm will have typical biofilms made up of the idiosyncratic microbiomes of the given insect species and the objects encountered in their direct environment, such as feeds, water, farm materials, and farm operators. To be on the safe side, farm biofilms should be sampled and evaluated in terms of risk assessment or simply be cleaned regularly. In addition, the site of frass in all experimental crates was found to be contaminated on day 14 after the start of this study. This indicated that, as expected, frass is another potential source of bacterial contamination because of microbial contents in the cricket gut [6,32,33,34]. An earlier report [18,33] found *E. coli* in cricket guts and frass, making the bacterium ubiquitous in humid areas of the production environment. These reports support the findings of *E. coli* contamination mainly on the water pipe, with some also found on the crate walls, the living cartons, the frass, and the surface of the crickets (Table 3). Microbial levels found in the frass samples in the current study were lower than those in other areas of the rearing crate. This is because the frass sampling site was a dry area on the rearing crate floor, where frass accumulated. Due to the behavior of crickets, they can leave droppings anywhere in the rearing crate, resulting in a high chance of spreading microbes. Regarding bacterial counts of *E. coli* at the sampling site for both two-spotted and house crickets, there were three samples detected for *E. coli* (Table 4) at concentrations of 20 (frass), 40 (water pipe), and 120 CFU/cm^2^ (frass) for two-spotted crickets, although the conventional method of bacterial culture could not find it, probably due to the better detection limit of the concentration measurement method. However, one sample tested positive for *E. coli* using the conventional method, while it tested negative for *E. coli* using the concentration method. This discrepancy might be due to the cylindrical shape of the water pipe, resulting in swabbing areas resembling thick lines along the length of the pipe. Although swabbing is a traditional method used to assert hygiene on surfaces in terms of species, the exact counts may vary according to the individual execution of even standardized swabbing methods. Consequently, the swabbed areas for each test method were selected on opposite sides, potentially containing different levels of *E. coli*. Not having encountered any *E. coli* in any of the animal samples suggested either a very low concentration or the total absence of that pathogen in both cricket species. Possibly, small amounts of *E. coli* were contained in the frass and then accumulated in the rearing area, especially drinking water supply equipment. Concerning the origin of bacterial contamination, it possibly originated from the gut microbes of crickets and from environmental contamination, including production resources, farm management, and working staff. The gut microbes of crickets included *Klebsiella* spp., *Citrobacter* spp., and *Enterobacter* spp. [6], which might be important sources of bacterial contamination in this study (Table 1), especially Klebsiella spp., which were detected in the highest proportion. Thus, the source of *E. coli* contamination in cricket rearing could be from the environment or humans. From the current study, tap water surroundings are a possible source of *E. coli* contamination, while the chance of human contamination is very low due to hand washing and laboratory clothing before and during work. However, *E. coli* is ubiquitous and associated with vertebrates, so it cannot be conclusively determined that the *E. coli* contamination in this study originated from humans. An in-depth molecular biology analysis should be conducted to possibly identify a human-related strain. In the meantime, crickets are omnivorous, and wild-ranging crickets could ingest contaminated feeds over time, such as plants or maggots that grow near vertebrate feces and/or water. The crickets in the current study were obtained from cricket eggs produced by a breeding cricket farm, which improves or maintains cricket genetics by including genetic lines from natural crickets and other breeding farms. While the contamination from wild-ranging crickets cannot be entirely excluded, it is less likely in this study as no plants or other feeds were imported to the cricket rearing units. Thus, cleaning the drinking water supply equipment with higher frequency would be an important management approach for cricket farms to lower the chance of bacterial contamination. Additionally, ensuring the sanitation of working staff both before and during work is a key strategy to reduce the chances of bacterial contamination, especially from *E. coli* and other bacteria commonly originating from humans. A concentration of *Salmonella* spp. was undetected from all collecting samples for both laboratory methods, in agreement with earlier reports [6,35,36,37]. According to a report by Liu [34], house crickets were unable to harbor *S. typhimurium* for longer than 6 days. This would be one explanation. However, a recent report by Praeg and Klammsteiner [33] showed that *Salmonella* spp. were detected in fresh frass of Jamaican field crickets (*Gryllus assimilis*; Fabricius, 1775).

From earlier reports [38,39], boiling crickets in a water bath at 96 °C for 5 min could eliminate all Enterobacteriaceae inhabiting the cricket gut. Boiling crickets for 5 min could also lower total aerobic counts [38,39,40]. These results agree with the findings of this study, as no *E. coli* was detected after boiling, and there was a lower proportion of bacterial contamination. Though there was no statistical difference (*p* > 0.05) for both studied cricket breeds, the proportion of the bacterial contamination of the cricket sample had a lower value (approximately 40% reduction for both two-spotted and house crickets) after boiling crickets for 5 min. This might not provide enough contact time with the heat from boiling, resulting in some bacteria inside the cricket body still surviving due to the protection provided by the abdominal organs against heat dissemination. There was a discrepancy in two reports indicating that total aerobic counts were reduced after boiling *Gryllodes sigillatus* for 5–10 min [41], but prolonging the boiling time from 5 to 10 min for larger crickets (*Brachytrupus* spp.) did not significantly influence the level of remaining bacteria [36]. This might be due to the different sizes of the crickets. A possible comparison is the reduction in *Listeria monocytogenes* and *Vibrio parahaemolyticus* in crabs, which required boiling at 79.5 °C for 20 min and 85 °C for 15 min. Thus, prolonging the boiling time from 5 min to 10–15 min for crickets might increase the likelihood of reducing total aerobic counts and many pathogenic bacteria. However, this also depends on the subsequent use, such as roasting or deep-frying. Ali et al. [42] showed the effectiveness of heat treatment for reducing the microbial counts in edible grasshoppers by boiling, sun-drying, and frying. Processing crickets by boiling and boiling–drying lowered the total aerobic plate counts for both *Gryllus assimilis* and house crickets when compared with unprocessed crickets (as crickets fresh and rinsed) [43]. These would be beneficial for using heat to control bacterial contamination. Concerning sporulating bacteria, spores are highly resistant and dormant structures that can survive extreme conditions such as heat, radiation, desiccation, and chemical exposure. A potential risk with edible insects is the presence of spore-forming bacteria [38]. *Bacillus* spp. often originate from soil and are not completely inactivated through heat treatment, resulting in spoilage or health risks when favorable conditions return for their germination and growth [4,44]. The absence of soil sources and lack of additional feed from plants would lower the chance of detecting *Bacillus* spp. in this study. Additionally, Bacillus cereus so far appears to come from dried products rather than fresh ones [45]. However, the situation is relatively new and under study, and more knowledge is necessary to evaluate the actual risk. Washing crickets by rinsing them in tap water would reduce only the bacteria inhabiting the external surface of crickets in accordance with the report of Fernandez-Cassi et al. [6], who found that pre-rinsing in water did not reduce bacterial contamination levels in house crickets. This would suggest that the animals are relatively clean from the outside, meaning that the animal-related, physico-chemical mechanisms worked.

In livestock production, good management practices, such as routinely cleaning water troughs, chlorinating or ozonating the water supply, and reducing fecal contamination, will greatly contribute to minimizing the spread and persistence of zoonotic bacteria on farms [46]. Cleaning and disinfectant application are crucial strategies to reduce the chances of opportunistic and pathogenic bacterial (nosocomial) infection in human [26,47,48,49,50,51] and animal hospitals [50,52,53]. Therefore, good routine management practices, physical cleaning, and avoiding moisture in cricket rearing environments would be beneficial to crickets and consumers. However, disinfectant application should be carried out with awareness of potential damage to reared crickets. However, all procedures in the current study followed Good Agricultural Practices (GAP) for Cricket Farm [54].

For antimicrobial agents used for resistance testing, several substance classes were applied to obtain an overview view of possible antimicrobial phenotype resistance. Penicillin (penicillin G) and aminopenicillin (amoxycillin, and ampicillin) are classified as β-lactam antimicrobials, while amoxicillin–clavulanic acid and cefalexin are classified as extended-spectrum β-lactam (ESBL) antimicrobials, while colistin is a polymyxin E. The mobilized colistin resistance (*mcr*) gene is produced by bacteria to resist colistin. Oxytetracycline and doxycycline are in a class of tetracycline. The quinolone class contains enrofloxacin and halquinal. Sulfamethoxazole–trimethoprim is in the class of sulphonamide and trimethoprim. Neomycin and gentamicin are classified as aminoglycosides. Lincomycin is in the class of lincosamide. Erythromycin and tiamulin are classified as macrolides. Multi-drug resistance (MDR) phenotypes were found in *E. coli* (Table 5 and Table 6). The common MDRs for *E. coli* in this study were substances from the beta-lactam (penicillin G, amoxycillin, and ampicillin), lincosamide (lincomycin), and macrolide (erythromycin, and tiamulin) categories. These common MDRs agreed with an earlier report [8] that found antimicrobial resistances to macrolide–lincosamide–streptogramin B (*erm*(A), *erm*(B), and *erm*(C)) and β-lactams (*blaZ*) in mesophilic aerobes isolated from Thailand’s edible arthropod species (giant water bugs, black ants, winged termite alates, rhino beetles, mole crickets, silk pupae, and black scorpions). The possible explanation for the MDR patterns in the current study might be sanitizer compounds as the quaternary ammonium compounds used in commercial farming provide cricket eggs in the current study. However, for *E. coli*, there were low resistance rates for tetracyclines (oxytetracycline) and quinolone (halquinol) and no resistance to colistin, doxycycline, enrofloxacin, sulfamethoxazole–trimethoprim, neomycin, or gentamicin. Presumably, the low rate and absence of antimicrobial resistance were good signs. In the past, rearing crickets in Thailand were always fed commercial chicken diets [55], containing possibly several antimicrobial agents [13,56]. However, mass cricket rearing [5] has applied an insect feed without antimicrobial agents for the past 5 years. This might be reflected by either the absence or low rate of antimicrobial resistance in crickets for some antimicrobial agents in the current study, while there was still high MDR in chicken or its products from Thailand, as reported in the past few years [57,58,59]. For the detection of β-lactam resistance, the EFSA Panel concluded that ESBLs may be defined as plasmid-encoded enzymes found in the Enterobacteriaceae, frequently in *E. coli* and *Klebsiella pneumoniae*, that confer resistance to a variety of β-lactam antimicrobials, including penicillin; second-, third-, and fourth-generation cephalosporins; and monobactams [8]. The macrolide–lincosamide–streptogramin B (MLSB) and *tet*(G) resistance genes could be transferred among bacteria via lateral gene transfer [60]. Furthermore, antimicrobial-resistant bacteria were transferred to eggs, larvae, pupae, and finally, the next generation of adult flies [16]. Regarding antimicrobial-resistant genes in house crickets, a high prevalence of genes conferring resistance to tetracycline [tet(M), tet(O), tet(K), tet(S)] was observed, together with the presence of genes conferring resistance to erythromycin [erm(B), erm(C)], beta-lactams (blaZ and mecA), and aminoglycosides [aac(6′)-Ie aph(2″)-Ia] [7]. Thus, further investigation concerning edible crickets should be conducted and pay more attention to antimicrobial resistance as little remains known about it. When considering the implications of antimicrobial treatment, it is well known that improper use can lead to antimicrobial resistance, a problem observed in both livestock production and companion animals (i.e., [61,62,63]). Currently, we strive to minimize antibiotic use and explore alternative treatments. Applying antibiotics to insects poses a direct threat to a supposedly well-balanced gut microbiome, potentially killing bacteria that are essential for proper digestion in insects. There is currently no standardized framework addressing the antibiotic treatment of farm insects. Therefore, much more research needs to be conducted before corresponding steps can be taken.

## 5. Conclusions

This study investigated bacterial contamination in the production chains of two-spotted (*G. bimaculatus*) and house (*A. domesticus*) crickets. There were 35- and 42-day periods for the rearing of two-spotted and house crickets, respectively. It was found that the majority of the detected bacteria were common bacteria that could be opportunistic pathogens, such as *Klebsiella* spp. and *Enterobacter*, which are mainly found in prepared drinking water and the water pipes of drinking water supply equipment, which probably contain potential sources of contamination with cricket frass. The presence of *E. coli* was mainly found on the water pipes, but *Salmonella* spp. could not be detected in the current study. Boiling crickets in the harvesting process showed lower values for the contaminated proportion. Quantifying *E. coli* and *Salmonella* spp. indicated the contamination of *E. coli* near the water pipe and the frass of two-spotted crickets, while undetectable for *Salmonella* spp. for both two-spotted and house crickets. The antimicrobial resistance of isolated *E. coli* mainly comprised penicillin G, amoxicillin, ampicillin, erythromycin, lincomycin, and tiamulin. Therefore, good farm management with proper sanitation practices (such as cleaning and keeping the environment dry), as well as boiling crickets during the harvesting process, may help ensure the safety of edible cricket production.

## Figures and Tables

**Table 1:** Overall bacterial isolations from submitted samples at the sampling site in the current experiment (values of 0 in this table represent the count number of samples in which bacteria could not be detected).

Sampling Site			Bacterial Isolations									
	Summited Sample	No Isolation of Bacteria	*Achromobacter* spp.	*Citrobacter* spp.	*Enterobacter* spp.	*Klebsiella* spp.	*Pasteurella aerogenes*	*Proteus* spp.	*Pseudonomas aeruginosa*	*Escherichia coli*	*Salmonella* spp.	Total Isolation
Input factor												
Cricket feed	10	10	0	0	0	0	0	0	0	0	0	0
Drinking water	10	4	1	0	2	5	1	0	0	0	0	9
Staff hands (before working)	5	5	0	0	0	0	0	0	0	0	0	0
Staff hands (after working)	5	4	1	0	0	0	0	0	0	0	0	1
Total	30	23	2	0	2	5	1	0	0	0	0	10
% of submitted samples		76.7	6.7	0.0	6.7	16.7	3.3	0.0	0.0	0.0	0.0	33.30
% of bacterial isolates			20.0 ^ab^	0.0 ^a^	20.0 ^ab^	50.0 ^b^	10.0 ^ab^	0.0 ^a^	0.0 ^a^	0.0 ^a^	0.0 ^a^	100.0
Two-spotted crickets:												
Rearing environment												
D1	40	32	0	1	5	8	0	0	0	2	0	16
D14	40	12	0	0	6	27	0	0	0	2	0	35
D28	40	6	0	0	9	32	1	0	2	3	0	47
D35	40	0	1	0	8	39	0	0	0	0	0	48
Total	160	50	1	1	28	106	1	0	2	7	0	146
% of submitted samples		31.3	0.6	0.6	17.5	66.3	0.6	0.0	1.3	4.4	0.0	91.3
% of bacterial isolates			0.7 ^abA^	0.7 ^ab^	19.2 ^c^	72.6 ^d^	0.7 ^ab^	0.0 ^a^	1.4 ^a^	4.8 ^a^	0.0 ^a^	100.0
House crickets:												
Rearing environment												
D1	40	32	0	0	6	8	1	0	0	0	0	15
D14	40	16	0	0	2	21	1	0	0	3	0	27
D28	40	13	0	0	6	25	0	0	4	2	0	37
D35	40	2	4	1	7	35	0	0	0	2	0	49
D42	35	1	10	4	9	30	0	2	2	1	0	59
Total	195	64	14	5	30	119	2	2	6	8	0	186
% of submitted samples		32.8	7.2	2.6	15.4	61.0	1.0	1.0	3.1	4.1	0.0	95.4
% of bacterial isolates			7.5 ^cB^	2.7 ^bc^	16.1 ^d^	64.0 ^e^	1.1 ^ab^	1.1 ^ab^	3.2 ^bc^	4.3 ^bc^	0.0 ^a^	100.0
Two-spotted crickets:												
Harvesting crickets												
Harvested crickets	8	0	0	0	2	8	0	0	0	0	0	10
Rinsed crickets	8	0	0	0	2	8	0	0	0	0	0	10
Boiled crickets	8	3	0	0	0	5	0	0	0	0	0	5
Total	24	3	0	0	4	21	0	0	0	0	0	25
% of submitted samples		12.5	0.0	0.0	16.7	87.5	0.0	0.0	0.0	0.0	0.0	104.2
% of bacterial isolates			0.0 ^a^	0.0 ^a^	16.0 ^a^	84.0 ^bB^	0.0 ^a^	0.0 ^a^	0.0 ^a^	0.0 ^a^	0.0 ^a^	100.0
House crickets:												
Harvesting crickets												
Harvested crickets	7	0	2	2	4	7	0	1	1	0	0	15
Rinsed crickets	7	0	0	2	4	7	0	1	1	0	0	14
Boiled crickets	7	3	0	1	2	4	0	0	0	0	0	10
Total	21	3	2	5	10	18	0	2	2	0	0	39
% of submitted samples		14.3	9.5	23.8	47.6	85.7	0.0	9.5	9.5	0.0	0.0	185.7
% of bacterial isolates			5.1 ^a^	12.8 ^ab^	25.6 ^bc^	46.2 ^cA^	0.0 ^a^	5.1 ^a^	5.1 ^a^	0.0 ^a^	0.0 ^a^	100.0

^abcde^ Proportions having different superscripts within the same row for the proportion of contaminated bacterial isolates are significantly different (*p* < 0.05). ^AB^ Proportions having different superscripts within the same column for comparing between experimental cricket species are significantly different (*p* < 0.05).

**Table 2:** The proportion of overall bacterial isolations at the sampling site varied with time of rearing and harvested processing for both two-spotted and house crickets in experimental unit (*n*) (values of 0 in this table represent the count number of samples in which bacteria could not be detected).

Item	Presence of Bacterial Isolate for Two-Spotted Crickets			Item	Presence of Bacterial Isolate for House Crickets		
	Uncontaminated Crates (*n*)	Contaminated Crates (*n*)	Percentage *^,^** (%)		Uncontaminated Crates (*n*)	Contaminated Crates (*n*)	Percentage *^,^** (%)
Water pipe				Water pipe			
D1	0	8	100.0	D1	0	8	100.0
D14	0	8	100.0	D14	0	8	100.0
D28	0	8	100.0	D28	0	8	100.0
D35	0	8	100.0	D35	0	8	100.0
				D42	0	7	100.0
Crate wall				Crate wall			
D1	8	0	0.0 ^a^	D1	8	0	0.0 ^a^
D14	8	0	0.0 ^a^	D14	7	1	12.5 ^ab^
D28	3	5	62.5 ^b^	D28	6	2	25.0 ^abc^
D35	0	8	100.0 ^b^	D35	2	6	75.0 ^c^
				D42	1	6	85.7 ^cd^
Living cartons				Living cartons			
D1	8	0	0.0 ^a^	D1	8	0	0.0 ^a^
D14	0	8	100.0 ^b^	D14	5	3	37.5 ^ab^
D28	1	7	87.5 ^b^	D28	3	5	62.5 ^bc^
D35	0	8	100.0 ^b^	D35	0	8	100.0 ^c^
				D42	0	7	100.0 ^c^
Frass				Frass			
D1	8	0	0.0 ^a^	D1	8	0	0.0 ^a^
D14	0	8	100.0 ^b^	D14	0	8	100.0 ^b^
D28	0	8	100.0 ^b^	D28	0	8	100.0 ^b^
D35	0	8	100.0 ^b^	D35	0	8	100.0 ^b^
				D42	0	7	100.0 ^b^
Cricket surface				Cricket surface			
D1	8	0	0.0 ^a^	D1	8	0	0.0 ^a^
D14	4	4	50.0 ^ab^	D14	4	4	50.0 ^ab^
D28	2	6	75.0 ^b^	D28	4	4	50.0 ^ab^
D35	0	8	100.0 ^b^	D35	0	8	100.0 ^b^
				D42	0	7	100.0 ^b^
Harvesting crickets				Harvesting crickets			
Harvested	0	8	100.0	Harvested	0	7	100.0
Rinsed	0	8	100.0	Rinsed	0	7	100.0
Boiled	3	5	62.5	Boiled	3	4	57.1

* Percentages calculated from the equation of Percentage = Contaminated crates × 100/(Uncontaminated crates + Contaminated crates. ** Multiple Chi-square is applied for comparing each sampling site or each process in harvesting crickets. ^abc^ Proportions having different superscripts within the same column for each sampling site or each process in harvesting crickets are significantly different (*p* < 0.05).

**Table 3:** Distribution for the number of isolations of *E. coli* found on sampling site at different cricket ages (values of 0 in this table represent the count number of samples in which bacteria could not be detected).

Sampling Site	Number of *E. coli* Isolations										Proportion of Positive *E. coli* Isolates
	Two-Spotted Cricket				House Cricket					Total	
	D1	D14	D28	D35	D1	D14	D28	D35	D42		
Water pipe	0	2	1	0	0	2	1	1	1	8	53.3 ^B^
Crate wall	0	0	1	0	0	0	0	0	0	1	6.7 ^A^
Living cartons	0	1	1	0	0	0	0	0	0	2	13.3 ^AB^
Frass	0	0	0	0	0	0	0	1	0	1	6.7 ^A^
Cricket surface	0	1	0	0	0	1	1	0	0	3	20.0 ^AB^
Total	0	4	3	0	0	3	2	2	1	15	100.0

^AB^ Proportions having different superscripts within the same column are significantly different (*p* < 0.05).

**Table 4:** Contamination levels of *E. coli* and *Salmonella* spp. at the sampling site for both two-spotted and house crickets in the current experiment (colony-forming unit = CFU).

Item	Bacterial Contamination Level ^β^	
	*E. coli*	*Salmonella* spp.
Cricket feed	<20 CFU/cm^2^ (*n* = 2)	<20 CFU/cm^2^ (*n* = 2)
Drinking water	<20 CFU/cm^2^ (*n* = 2)	<20 CFU/cm^2^ (*n* = 2)
Hands (before working)	<20 CFU/cm^2^ (*n* = 1)	<20 CFU/cm^2^ (*n* = 1)
Hands (after working)	<20 CFU/cm^2^ (*n* = 1)	<20 CFU/cm^2^ (*n* = 1)
	Two-spotted crickets	
Rearing environment		
Water pipe	<20 CFU/cm^2^ (*n* = 7), 40 CFU/cm^2^ (*n* = 1),	<20 CFU/cm^2^ (*n* = 8)
Crate wall	<20 CFU/cm^2^ (*n* = 8)	<20 CFU/cm^2^ (*n* = 8)
Living cartons	<20 CFU/cm^2^ (*n* = 8)	<20 CFU/cm^2^ (*n* = 8)
Frass	<20 CFU/cm^2^ (*n* = 6), 120 CFU/cm^2^ (*n* = 1), 20 CFU/cm^2^ (*n* = 1)	<20 CFU/cm^2^ (*n* = 8)
Cricket surface	<20 CFU/cm^2^ (*n* = 8)	<20 CFU/cm^2^ (*n* = 8)
Harvesting crickets		
Harvested crickets	<10 CFU/g (*n* = 8)	<10 CFU/g (*n* = 8)
Rinsed crickets	<10 CFU/g (*n* = 8)	<10 CFU/g (*n* = 8)
Boiled crickets	<10 CFU/g (*n* = 8)	<10 CFU/g (*n* = 8)
	House crickets	
Rearing environment		
Water pipe	<20 CFU/cm^2^ (*n* = 8)	<20 CFU/cm^2^ (*n* = 8)
Crate wall	<20 CFU/cm^2^ (*n* = 8)	<20 CFU/cm^2^ (*n* = 8)
Living cartons	<20 CFU/cm^2^ (*n* = 8)	<20 CFU/cm^2^ (*n* = 8)
Frass	<20 CFU/cm^2^ (*n* = 8)	<20 CFU/cm^2^ (*n* = 8)
Cricket surface	<20 CFU/cm^2^ (*n* = 8)	<20 CFU/cm^2^ (*n* = 8)
Harvesting crickets		
Harvested crickets	<10 CFU/g (*n* = 7)	<10 CFU/g (*n* = 7)
Rinsed crickets	<10 CFU/g (*n* = 7)	<10 CFU/g (*n* = 7)
Boiled crickets	<10 CFU/g (*n* = 7)	<10 CFU/g (*n* = 7)

^β^ The accepted microbial levels (total aerobic counts) in raw, processed, and packaged meat should be below 10^5^ CFU/g of meat, and not fit for human consumption when beyond 10^7^ CFU/g of meat. Cooked meat (cooking or post-processing contamination) for consumption should contain organisms below 10^2^ CFU/g of meat. *Campylobacter* spp., *E. coli*, and *Salmonella* spp. should not be detected in 25 g of meat [18].

**Table 5:** Antimicrobial resistance to *E. coli* at the sampling site for both two-spotted and house crickets in the current experiment (values of 0 in this table represent the count number of samples in which bacteria could not be detected).

Item	Numbers of Samples with Detected Antimicrobial Resistance to *E. coli* *															
	PNG	AMC	AMX	AMP	CEF	COL	OTC	DOX	ENR	HAL	SXT	NEO	GEN	LIN	ERY	TIA
Two-spotted crickets																
Water pipe	4	0	4	4	0	0	0	0	0	1	0	0	0	4	4	4
Crate wall	1	0	1	1	0	0	0	0	0	0	0	0	0	1	1	1
Living cartons	2	0	2	2	0	0	1	0	0	0	0	0	0	2	2	2
Cricket surface	1	0	1	1	0	0	0	0	0	0	0	0	0	1	1	1
Sub-total	8	0	8	8	0	0	1	0	0	1	0	0	0	8	8	8
House crickets																
Water pipe	4	0	4	4	0	0	0	0	0	0	0	0	0	4	4	4
Frass	1	0	1	1	0	0	0	0	0	0	0	0	0	1	1	1
Cricket surface	2	0	2	2	1	0	1	0	0	0	0	0	0	2	2	2
Sub-total	7	0	7	7	1	0	1	0	0	0	0	0	0	7	7	7
Total	15	0	15	15	1	0	2	0	0	1	0	0	0	15	15	15

* PNG = penicillin G, AMC = amoxicillin–clavulanic acid, AMX = amoxycillin, AMP = ampicillin, CEF = cefalexin, COL = colistin, OTC = oxytetracycline, DOX = doxycycline, ENR = enrofloxacin, HAL = halquinal, SXT = sulfamethoxazole–trimethoprim, NEO = neomycin, GEN = gentamicin, LIN = lincomycin, ERY = erythromycin, and TIA = tiamulin.

**Table 6:** Antibiograms of *E. coli* of isolated samples in the current study (values of 0 in this table represent the count number of samples in which bacteria could not be detected).

Pattern	Profile *	Number of Resistant Antimicrobial Agents	Number of Isolates		
			Two-Spotted Cricket	House Cricket	Total
1	PNG + AMX + AMP + LIN + ERY + TIA	6	6	5	11
2	PNG + AMX + AMP + LIN + ERY + TIA + OTC	7	1	1	2
3	PNG + AMX + AMP + LIN + ERY + TIA + CEF	7	1	0	1
4	PNG + AMX + AMP + LIN + ERY + TIA + HAL	7	0	1	1

* PNG = penicillin G, AMX = amoxycillin, AMP = ampicillin, CEF = cefalexin, OTC = oxytetracycline, HAL = halquinal, LIN = lincomycin, ERY = erythromycin, and TIA = tiamulin.

## Data Availability

The original contributions presented in the study are included in the article. Further inquiries can be directed to the corresponding author.
